# Emotional dispositions and intracerebral hemorrhage: a Mendelian Randomization insight

**DOI:** 10.3389/fgene.2024.1330682

**Published:** 2024-06-20

**Authors:** Tian Hou, Yipeng Xu, Aili Buaijier, Xuetao Yu, Yuchen Guo, Di Zhang, Peng Liu

**Affiliations:** Department of Rehabilitation, People's Hospital of Xinjiang Uygur Autonomous Region, Urumqi, Xinjiang, China

**Keywords:** stroke, intracerebral hemorrhage, emotion, worry, anxiety, Mendelian randomization, single nucleotide polymorphisms, risk factor

## Abstract

**Background:**

Intracerebral hemorrhage (ICH) is a severe form of stroke with high mortality and limited treatment options. While traditional risk factors like hypertension have been well-studied, the role of emotional states as acute triggers for ICH remains unclear. This study employs Mendelian Randomization (MR) to investigate the causal relationship between emotional traits of worry and anxiety and the incidence of ICH.

**Methods:**

We used a two-sample MR approach, leveraging summary-level data from genome-wide association studies (GWAS) for emotional traits and ICH. The primary analysis was conducted using the Inverse-Variance Weighted (IVW) method, supplemented by multiple sensitivity analyses including Maximum Likelihood and MR PRESSO methods.

**Results:**

Our MR analysis revealed a robust and significant causal relationship between the emotional trait “Worrier/anxious feelings” and ICH, supported by 195 instrumental variables (SNPs). The odds ratio (OR) was 2.98 (95% CI: 1.16, 7.61) with a *p*-value of 0.0229. Sensitivity analyses corroborated these findings, enhancing the reliability of our results. In contrast, other emotional traits such as “Nervous feelings” and “Sensitivity/hurt feelings” did not show significant associations, reinforcing the specificity of our primary finding.

**Conclusion:**

Our study provides compelling evidence for a causal relationship between the emotional traits of worry and anxiety and the incidence of ICH, offering a new dimension in our understanding of this devastating condition and paving the way for more nuanced risk stratification and preventive strategies.

## Introduction

Intracerebral hemorrhage (ICH), a non-traumatic form of spontaneous intracranial bleeding, constitutes the most severe and least amenable variant of stroke (Cordonnier et al., 2018). Accounting for approximately 10%–15% of all strokes, ICH presents a disproportionately high mortality rate, with nearly 40% of affected individuals succumbing within the first month (Fujii et al., 1994; Schrag and Kirshner, 2020). This condition afflicts an estimated two million individuals worldwide annually, eclipsing other intracranial haemorrhage subtypes such as subarachnoid and isolated intraventricular haemorrhages in both prevalence and gravity (Krishnamurthi et al., 2013). The societal ramifications are considerable, extending beyond mortality to include long-term disability and substantial healthcare expenditures (Ruiz-Sandoval et al., 1999).

While substantial strides have been made in mitigating the incidence of ICH through the rigorous management of established risk factors such as hypertension, smoking, and diabetes, it appears that we have reached an epidemiological plateau in these episode preventive endeavours (O'Donnell et al., 2010; Lovelock et al., 2007). To transcend this impasse and further attenuate the episode incidence of ICH, there is an emergent imperative to unearth novel risk determinants and acute triggers (Elkind, 2007). The concept of “triggers” delineated as acute precipitants capable of instigating an ICH episode, is increasingly being accorded pivotal importance. The elucidation of such triggers holds the promise of not only enhancing risk stratification algorithms but also fortifying predictive models and refining both short-term and long-term preventive strategies. Emerging evidence is starting to suggest that emotional states may influence and act as triggers for ICH (Aalbaek et al., 2017; Prasad et al., 2020; Smyth et al., 2022).

Current research on the triggering factors of ICH predominantly employs the case-crossover study design (Haynes, 2011; Liu et al., 2023). This approach is favored for its ability to capture transient exposures that may precipitate acute events like ICH (Maclure, 1991). Additionally, the design inherently controls for stable confounders by allowing each individual to serve as their own control, thereby enhancing the internal validity of the study. The case-crossover design is also more feasible and efficient compared to randomized controlled trials (RCTs) or cohort studies, especially when focusing on acute triggers.

However, this design is not without its limitations. One major drawback is the potential for residual confounding from time-varying factors that are not accounted for in the study. Furthermore, case-crossover studies often lack the rigor of higher-level evidence that could be obtained from controlled or prospective studies. This makes it challenging to definitively establish causality between potential triggers and ICH. Given the severity and complexity of ICH, there is an urgent need for high-quality evidence to conclusively identify its triggers. This would require innovative research methodologies that can eliminate all forms of confounding, thereby providing more reliable and actionable insights into the prevention of ICH.

Mendelian Randomization (MR) offers a robust alternative to traditional epidemiological methods for causal inference, minimizing confounding and reverse causation (Skrivankova et al., 2021; Verbanck et al., 2018; Thanassoulis and O'Donnell, 2009). MR has been widely applied in cardiovascular and cerebrovascular research, including studies on ICH (Larsson et al., 2019; Titova et al., 2020; Xiao et al., 2022; Yu et al., 2022; Wang et al., 2023). This makes it an ideal tool for exploring the yet-to-be-established causal relationship between various emotional states and ICH. Given the critical need for effective ICH prevention, MR could provide the high-quality evidence required to inform better preventive strategies.

In light of these considerations, the objective of this investigation is to employ MR analysis as a rigorous methodological approach to furnish elevated tiers of evidence, meticulously eradicate confounding variables, and discern the specific emotional states causally implicated in ICH.

## Materials and methods

### Data sources

**FIGURE 1:**
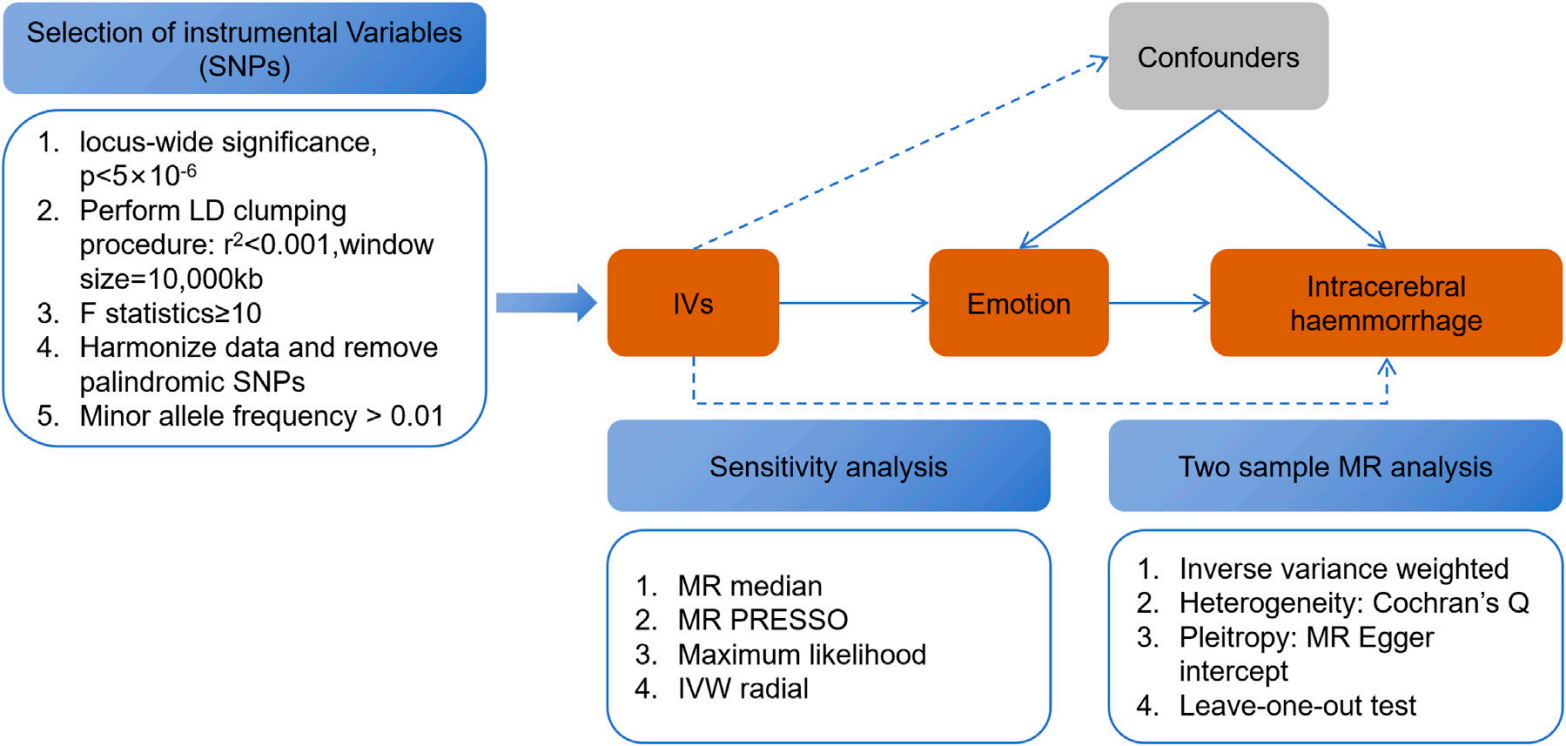
Flowchart of the present MR study and major assumptions.

The methods employed in this study are comprehensively illustrated in Figure 1, a flowchart detailing the entire analytical process. To investigate the causal association between emotional dispositions and Intracerebral Hemorrhage (ICH) incidence, we utilized summary-level data extracted from Genome-Wide Association Studies (GWAS). The data concerning emotional dispositions were sourced from the MRC-IEU consortium, which encompasses European populations, and includes a vast array of 9,851,867 single nucleotide polymorphisms (SNPs). GWAS information pertaining to feelings can be accessed on the website (https://gwas.mrcieu.ac.uk/), with the following identifiers: Happiness, fed-up feelings, worrier/anxious feelings, guilty feelings, nervous feelings, sensitivity/hurt feelings, miserableness, and loneliness/isolation (Table 1). It is worth noting that, except for Happiness, which is classified as categorical ordered, the remaining feelings are represented as binary variables. These variables were derived from GWAS pipeline using phesant-derived variables from UK Biobank (Supplementary Table S1). Conversely, ICH-related data were derived from the FinnGen study, as identified by the GWAS ID: finn-b-I9_ICH, comprising 1,687 cases and 2,01,146 controls, and encapsulating a total of 16,380,393 SNPs (refer to Supplementary Table S1 for details).

**TABLE 1:** GWAS summary-level data of emotion.

id	Trait	Ncase	year	Author	Consortium	Sex	Population	Sample_size	n_control	nsnp
ukb-b-9981	Sensitivity/hurt feelings	249799	2018	Ben Elsworth	MRC-IEU	Males and Females	European	449419	199620	9851867
ukb-b-4062	Happiness	NA	2018	Ben Elsworth	MRC-IEU	Males and Females	European	152348	NA	9851867
ukb-b-10093	Tense/'highly strung'	78408	2018	Ben Elsworth	MRC-IEU	Males and Females	European	447961	369553	9851867
ukb-b-6519	Worrier/anxious feelings	255812	2018	Ben Elsworth	MRC-IEU	Males and Females	European	450765	194953	9851867
ukb-b-20544	Nervous feelings	106635	2018	Ben Elsworth	MRC-IEU	Males and Females	European	450700	344065	9851867
finn-b-I9_ICH	Intracerebral hemmorrhage	1,687	2021	NA	NA	Males and Females	European	NA	201146	16380393

### Instrumental variable (IV) selection

The instrumental variables were meticulously selected adhering to a set of stringent criteria. Initially, SNPs were filtered based on a locus-wide significance threshold of *p* < 5 × 10^−6^. This preliminary step was followed by linkage disequilibrium (LD) clumping, executed with a rigorous threshold of less than 0.001 and a window size of 10,000 kb to ensure robust clump selection. Subsequently, only the SNPs with F-statistics of 10 or higher were retained for further analysis to guarantee robust instrument strength. A thorough data harmonization was performed to ascertain consistency across datasets, during which palindromic SNPs were excluded to prevent any allelic ambiguity. Lastly, SNPs with a minor allele frequency exceeding 0.01 were incorporated into the analysis to ensure adequate population representation.

### Statistical analysis

The MR computations were orchestrated using the adept TwoSampleMR package in R. Our primary causal estimates were diligently derived employing the IVW method. A gamut of sensitivity analyses was conducted to ascertain the robustness of our findings, utilizing an array of techniques including MR Median, MR PRESSO, Maximum Likelihood, and IVW Radial.

Heterogeneity among the instrumental variables was quantified employing Cochran’s Q test, while pleiotropy was meticulously assessed through the MR-Egger intercept to ensure the integrity of the MR assumptions. A rigorous leave-one-out analysis was conducted to validate the robustness of the results, ensuring that our findings were not unduly influenced by any single SNP.

For a robust statistical analysis, summary data were harmonized between the exposure and outcome datasets to ensure consistency in the SNP alleles and effect estimates. A comprehensive examination of the datasets was undertaken to identify and rectify potential mismatches or discrepancies, thereby ensuring the integrity of the analysis. The significance level was meticulously set at *p* < 0.05 for all statistical tests. All analyses were conducted using R software, and results were presented with 95% confidence intervals, providing a rigorous statistical framework for interpreting the findings.

## Results

### Instrumental variable selection and analysis

Instrumental variables (IVs) were selected based on stringent criteria, including a locus-wide significance threshold of 5 × 10^−6^ and a linkage disequilibrium *r* ^2^ < 0.001. After matching these IVs with the SNPs associated with the outcome of ICH, we identified five emotional traits for further analysis. Using the IVW method, the number of IVs for each emotional trait were as follows: 195 for ukb-b-6519, 162 for ukb-b-20544, 147 for ukb-b-9981, 140 for ukb-b-10093, and 45 for ukb-b-4062. Comprehensive results are presented in Supplementary Table S1.

### Mendelian randomization

**FIGURE 2:**
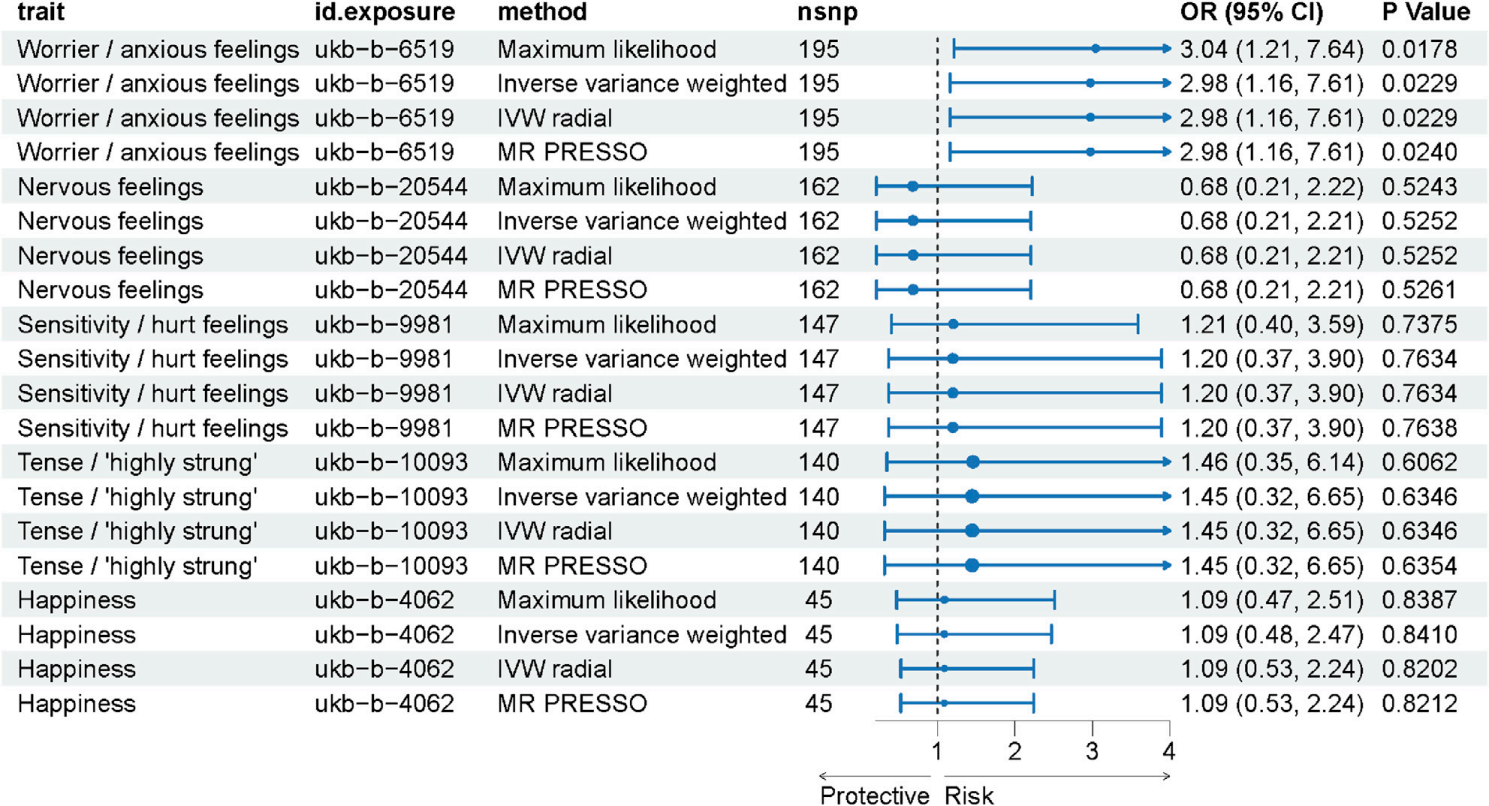
Forest Plot Representing MR Analysis Results. This figure displays the results of the Mendelian Randomization (MR) analysis, showcasing the causal relationships between various emotional traits and the incidence of intracerebral hemorrhage (ICH).

In our Mendelian Randomization analysis, we focused on elucidating the causal relationships between specific emotional traits and ICH (Figure 2). For the trait “Worrier/anxious feelings,” represented by 195 SNPs, a significant association with ICH was observed across multiple methods, detailed in Supplementary Table S2. Utilizing the IVW method as our primary analysis (Table 2), we observed a significant association with a Beta of 1.09037, a standard error of 0.47925, yielding an odds ratio (OR) of 2.98 (95% CI: 1.16, 7.61) and a *p*-value of 0.0229. This result was substantiated by the Maximum Likelihood method (Beta±SE: 1.09037 ± 0.47925, *p* = 0.0229) and the MR PRESSO method (Beta±SE: 1.09037 ± 0.47925, *p* = 0.0239). Although the Weighted Median method did not yield a significant association (Beta±SE: 0.58832 ± 0.66703, *p* = 0.3778), these findings collectively suggest a significant causal relationship between the trait “Worrier/anxious feelings” and ICH.

**TABLE 2:** Results of Mendelian Randomization analysis.

Trait	id.exposure	nsnp	b	se	OR (95%CI)	Pval
Worrier/anxious feelings	ukb-b-6519	195	1.090371261	0.479251326	2.98 (1.16, 7.61)	0.022896607
Nervous feelings	ukb-b-20544	162	−0.379753879	0.597745562	0.68 (0.21, 2.21)	0.525226094
Sensitivity/hurt feelings	ukb-b-9981	147	0.181089153	0.601476757	1.20 (0.37, 3.90)	0.763357889
Tense/'highly strung'	ukb-b-10093	140	0.369633867	0.77784928	1.45 (0.32, 6.65)	0.634644529
Happiness	ukb-b-4062	45	0.084000228	0.418755896	1.09 (0.48, 2.47)	0.841015475

In contrast, other emotional traits such as “Nervous feelings,” represented by 162 SNPs, and “Sensitivity/hurt feelings,” represented by 147 SNPs, did not show significant associations, with *p*-values consistently above 0.05. These findings suggest that among the emotional traits studied, “Worrier/anxious feelings” may have a significant causal relationship with ICH, thereby warranting further investigation into potential causal pathways.

### Heterogeneity and pleiotropy assessment

**FIGURE 3:**
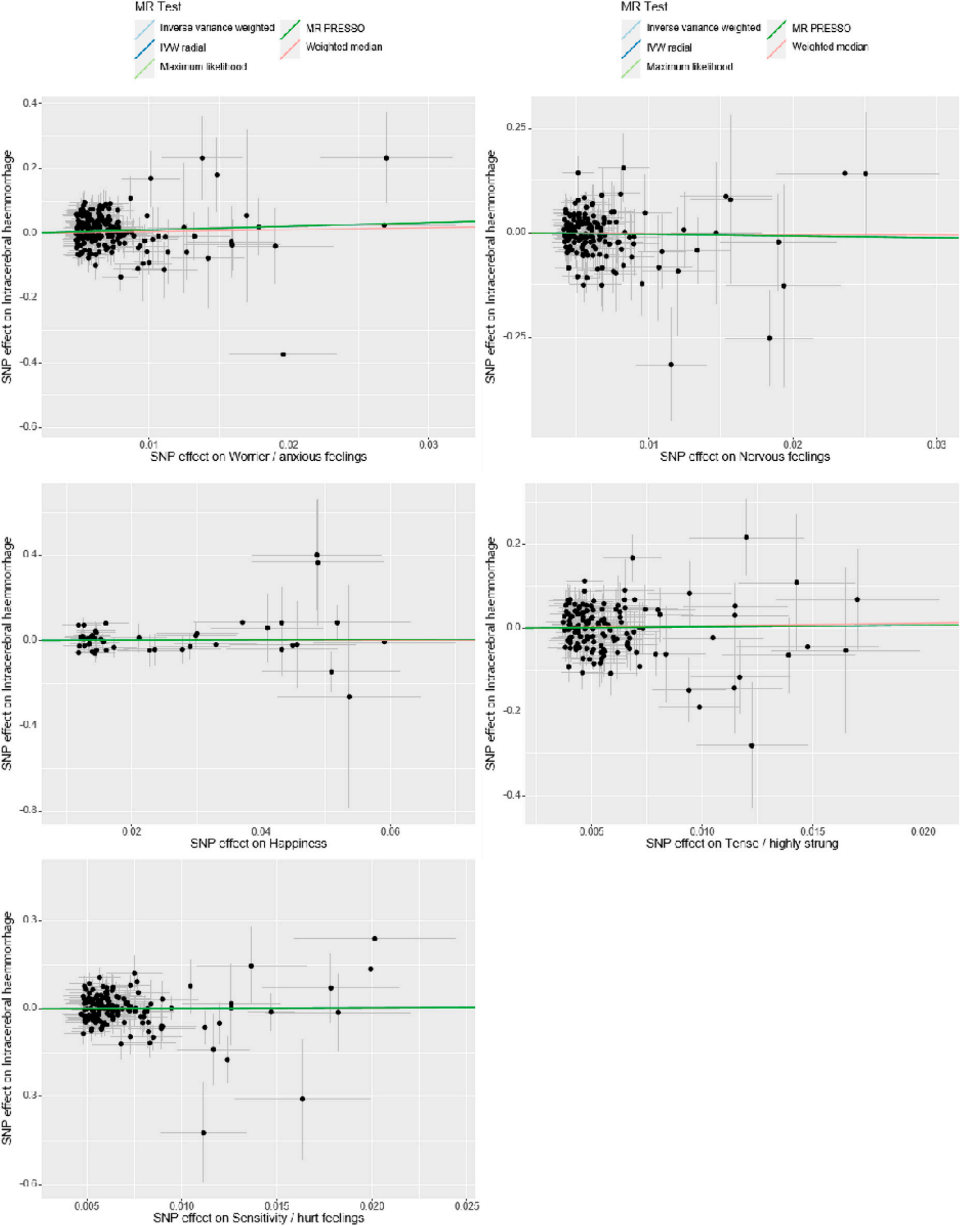
Sensitivity Analysis. The *x*-axis represents the effect size between the SNPs and the exposure, while the *y*-axis represents the effect size between the SNPs and the outcome. Black dots represent the effect values of SNPs with both exposure and outcome, and the solid line represents the causal association between the variables and the outcome. Different colors represent different MR methods used in the analysis.

**FIGURE 4:**
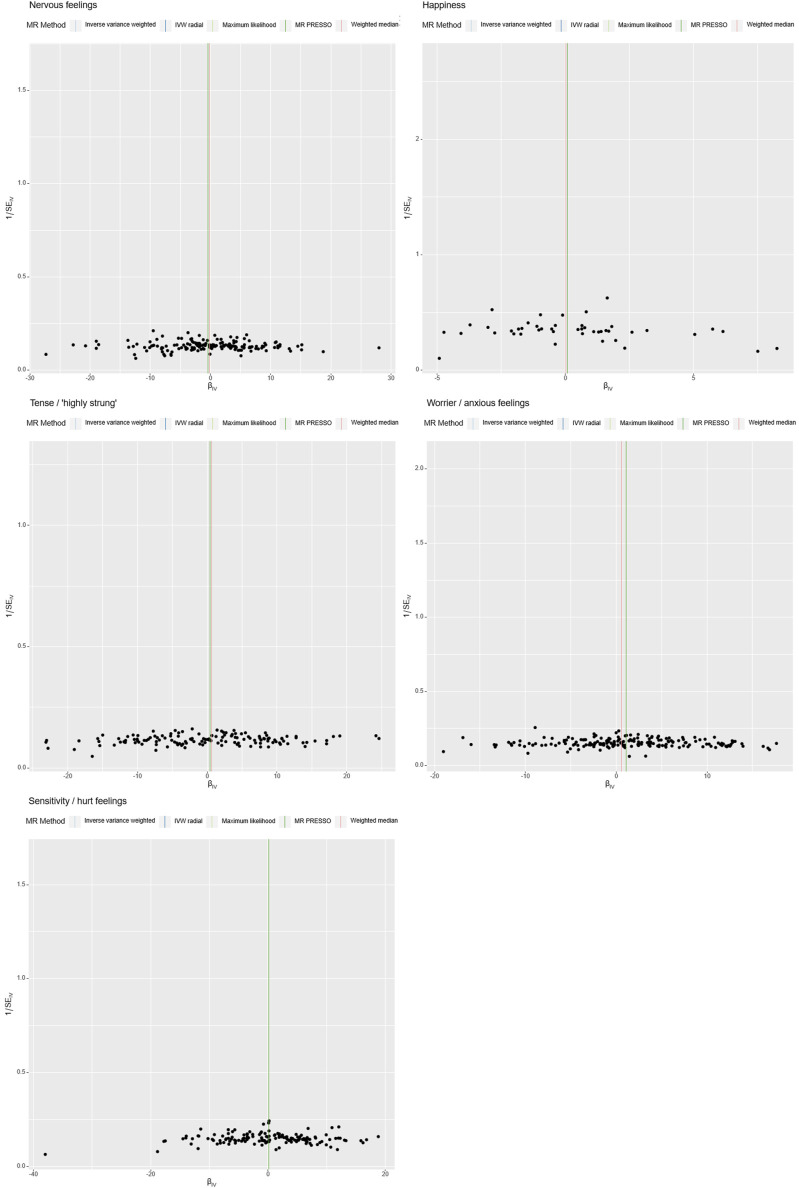
Heterogeneity Analysis. Instrumental variables from different analytical platforms, experiments, and populations may exhibit heterogeneity, thereby affecting the results of the Mendelian Randomization analysis. Heterogeneity is assessed using both IVW (Inverse-Variance Weighted) and MR-Egger tests, with a *p*-value <0.05 indicating the presence of heterogeneity in the study.

In our Mendelian Randomization analysis, we employed additional tests to assess the heterogeneity and pleiotropy of our instrumental variables and to explore potential sources of bias (Table 3, Figures 3, 4). For the trait “Worrier/anxious feelings,” the Egger intercept was 0.0164 with a standard error of 0.0129, resulting in a non-significant *p*-value of 0.204.

**TABLE 3:** Evaluation of heterogeneity and directional pleiotropy.

Trait	id.exposure	egger_intercept	se_intercept	pval_intercept	Q	Q_df	Q_pval
Worrier/anxious feelings	ukb-b-6519	0.016421352	0.012890328	0.204220826	209.9018966	194	0.206146558
Nervous feelings	ukb-b-20544	0.016161252	0.012770342	0.207521152	163.8892861	161	0.421847977
Sensitivity/hurt feelings	ukb-b-9981	0.02033644	0.016096692	0.208475742	177.9338693	146	0.037048571
Tense/'highly strung'	ukb-b-10093	0.018743178	0.015378604	0.225005973	163.7010008	139	0.074756271
Happiness	ukb-b-4062	0.005094174	0.017413373	0.771277913	34.26517997	44	0.853927723

Specially, “Sensitivity/hurt feelings” exhibited significant heterogeneity (Q = 177.93, df = 146, *p* = 0.037) but a non-significant Egger intercept of 0.0203 (SE = 0.0161, *p* = 0.208). Conversely, the traits “Nervous feelings,” “Tense/‘highly strung’,” and “Happiness” yielded non-significant results for both the Egger intercept and Cochran’s Q, thereby indicating a lack of both heterogeneity and pleiotropy. These findings collectively imply that, with the exception of “Sensitivity/hurt feelings,” the emotional traits under investigation do not exhibit significant biases and are not causally linked to ICH.

**FIGURE 5:**
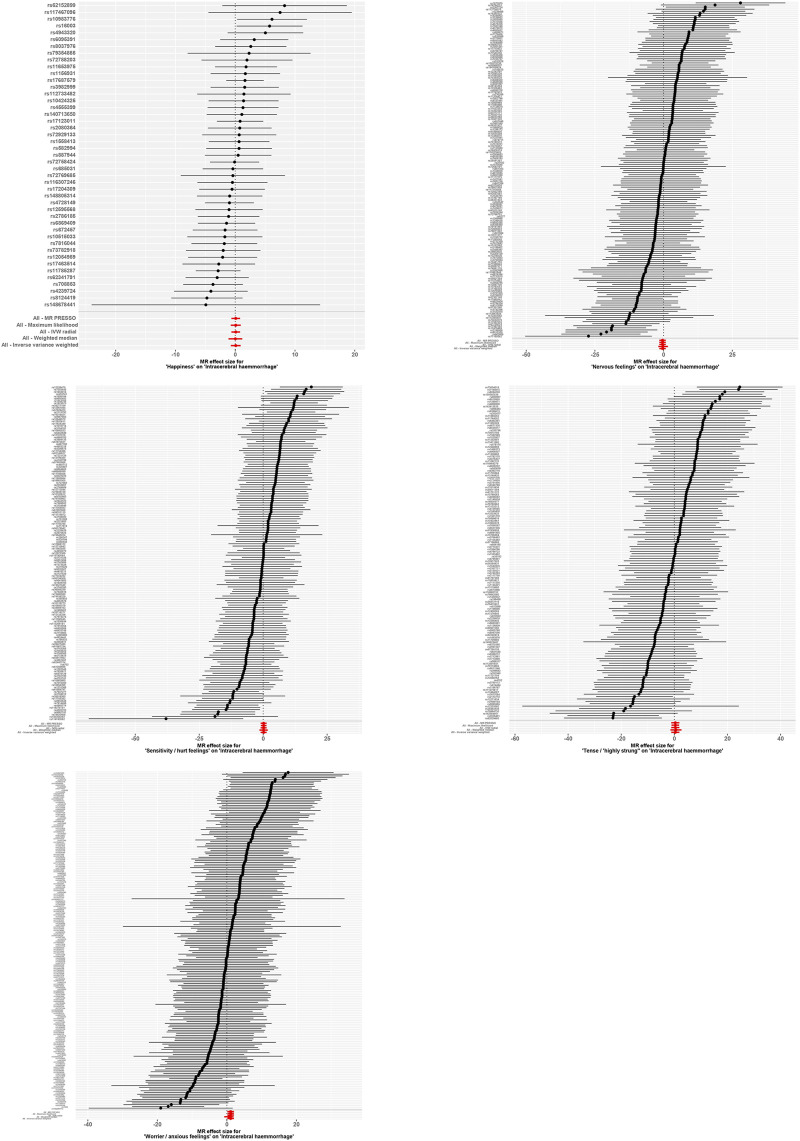
Forest Plot with Leave-One-Out Sensitivity Test. This figure further conducts a leave-one-out sensitivity test to check whether the causal estimates are driven by any single SNP. It reveals a consistent positive correlation between the genetic predictors and the risk of the outcome.

Further analyses were conducted using a leave-one-out sensitivity test to ascertain whether the causal estimates were driven by any single SNP in Figure 5. The test revealed a consistent positive correlation between the genetic predictors and the risk of the outcome. Heterogeneity that could potentially affect the Mendelian randomization results, arising from different analytical platforms, experiments, or populations, was assessed using both IVW and MR-Egger tests. With *p* > 0.05 in both tests, our study indicated no significant heterogeneity.

## Discussion

Our MR study robustly demonstrated a significant causal relationship between the emotional trait “Worrier/anxious feelings” and ICH, supported by 195 instrumental variables (SNPs). Conversely, other emotional traits like “Nervous feelings,” “Sensitivity/hurt feelings,” and “Tense/'highly strung'” did not show significant associations, reinforcing the specificity of our primary finding. Additional tests for heterogeneity and pleiotropy further corroborated these results. For the first time, our Mendelian Randomization study provides compelling evidence that worry and anxiety can directly trigger ICH. This study adds a new dimension to our understanding of ICH triggers, potentially paving the way for more targeted preventive strategies.

In our study, the IVW, IVW Radial, Maximum Likelihood, and MR PRESSO methods all have *p*-values below 0.05, indicating a statistically significant association between the exposure and the outcome. In contrast, the Weighted Median method has a *p*-value greater than 0.05, suggesting a lack of statistical significance. The IVW method is commonly used in MR because it combines the effects of individual SNPs weighted by their inverse variance, assuming no horizontal pleiotropy, where a genetic variant affects the outcome through pathways other than the exposure of interest (Lin et al., 2021). The IVW Radial variant accounts for outliers and handles potential pleiotropy better. The Weighted Median method offers a valid causal estimate even if up to 50% of the information comes from invalid instruments, but it is less efficient than IVW and may be influenced by outliers, which could explain its non-significant result in this case (Scholz, 1978). In this study, the IVW method serves as the primary analysis because it generally provides efficient and reliable estimates, assuming no substantial horizontal pleiotropy. The Weighted Median method differs in its assumptions and is less efficient. The other methods offer complementary analyses to address different assumptions and identify potential pleiotropy.

The quest for acute triggers in ICH is becoming increasingly pivotal, especially as traditional risk management approaches appear to have hit a ceiling in reducing incidence rates (Puy et al., 2023). Identifying novel, non-traditional risk factors or preventable triggers, including acute triggers, opens up new avenues for intervention. It allows for the development of more targeted and personalized prevention strategies, which could include behavioral modifications, psychological interventions, or even pharmacological treatments specifically designed to mitigate these triggers. Moreover, the discovery of such triggers could enhance risk stratification models, enabling clinicians to more accurately identify individuals at heightened risk of ICH. This, in turn, could lead to more timely and effective interventions, potentially reducing the incidence and severity of ICH in vulnerable populations. The prospects are promising, offering hope for significant advancements in ICH prevention and management.

The INTERSTROKE study, a comprehensive international case-control investigation, has identified the leading risk factors for stroke. Notably, two of the top ten risk factors—psychosocial stress and depression—are related to emotional states, thereby offering indirect relevance to our own research (O'Donnell et al., 2010). A specific component of the INTERSTROKE study focuses on the role of anger, emotional upset, and heavy physical exertion as acute triggers for stroke. The authors reported that instances of anger or emotional upset occurring within the hour preceding a stroke were relatively common and exhibited a notable association with ICH. This finding is consistent with previous small-scale case-crossover studies (Koton et al., 2004).

In practical terms, case-control and crossover studies are often the most feasible designs for investigating triggers or risk factors for ICH. These study designs are particularly suited for capturing acute triggers and short-term risk factors that may precipitate ICH events (Maclure and Mittleman, 2000). However, to yield credible results, these studies require a large scale, often necessitating substantial investment in terms of human, financial, and time resources. Despite these considerable efforts, the evidence level generated from case-control and crossover studies is not considered the highest, leaving room for further methodological advancements to provide more definitive conclusions.

In the absence of high-quality clinical evidence, our MR study provides a compelling case for a causal relationship between the emotional traits of worry and anxiety and the incidence of ICH. This underscores the importance of considering emotional states as potential acute triggers, thereby offering a nuanced approach to further reducing ICH incidence.

Furthermore, clinical studies have established a link between anxiety or other negative emotions and ICH, although they do not elucidate directional causality. Animal studies, however, provide evidence that ICH can lead to negative emotional states. The study on thalamic ICH highlights its role in triggering Central Post-Stroke Pain (CPSP) and subsequent emotional comorbidities like anxiety and depression, suggesting therapeutic potential for stellate ganglion block (SGB) (Shi et al., 2023). Similarly, Zhu et al. also demonstrate that right hemispheric ICH in mice leads to changes in emotion-related behavior, adding another layer of complexity to the neurological and behavioral impact of ICH (Zhu et al., 2018). In contrast, our Mendelian Randomization study has the unique advantage of providing a unidirectional causal relationship between emotional states and ICH. This strengthens the case for emotional states as potential triggers for ICH and underscores the value of MR in advancing our understanding of these complex relationships.

Last in the list, previous research has robustly demonstrated that negative emotional states like anger can have a significant impact on cardiovascular systems, thereby increasing the risk of cardiovascular diseases (CVD) (Mittleman et al., 1995; Stalnikowicz and Tsafrir, 2002; Smyth et al., 2016). Given that CVD is a well-established major risk factor for ICH, the question arises whether the emotional traits directly trigger ICH or do so indirectly through their impact on CVD (Qureshi et al., 2009; Sheth, 2022). The intricacy of isolating the direct effects of emotional states on ICH is further complicated by the presence of numerous confounding factors in real-world settings. RCTs, although considered the gold standard for establishing causality, are particularly challenging to implement for studying acute triggers like ICH. Ethical considerations, logistical constraints, and the acute nature of the condition make RCTs nearly impractical for this line of research. Even if an RCT were feasible, it would still face the hurdle of controlling for a myriad of confounding variables, such as pre-existing CVD conditions, lifestyle factors, and medication use. Consequently, the challenge remains to design clinical studies that can effectively eliminate these confounders while still being both ethical and logistically feasible. In the absence of such high-level evidence, Mendelian Randomization serves as a valuable alternative for causal inference, albeit with its own limitations.

Our study, while pioneering in its identification of a causal relationship between the emotional traits of worry and anxiety and the incidence of ICH, is not devoid of limitations that warrant discussion. Primarily, the observational nature of the data, despite the methodological rigour afforded by MR, leaves room for residual confounding. Additionally, the paucity of SNPs serving as instrumental variables for certain emotional traits may compromise the statistical power of our analyses. The study’s external validity is also circumscribed by the demographic characteristics of the cohort, cautioning against overgeneralization. Moreover, the intricate nexus between emotional states, cardiovascular diseases, and ICH remains partially obscured, necessitating further research to elucidate potential intermediary mechanisms. It is also pertinent to note that while MR offers a higher echelon of evidence compared to traditional epidemiological methods, it does not supplant the gold standard of randomized controlled trials, which are logistically challenging in this domain. Lastly, the scope of our investigation was inherently constrained by the availability of suitable emotional traits within the genetic databases, limiting our ability to explore the full spectrum of negative emotions that may be implicated in ICH.

Our MR study could provide valuable insights into causal relationships but is subject to limitations. Pleiotropy, where genetic variants influence outcomes through multiple pathways, can confound results, necessitating sensitivity analyses. Population stratification, reflecting genetic diversity across populations, can bias MR results. Researchers should employ statistical techniques or focus on homogeneous populations to mitigate this bias. The strength of instrumental variables is crucial in MR. Weak instruments can lead to biased estimates or reduced statistical power, emphasizing the need for robust SNP selection. Temporal relationships can be ambiguous, especially in cases involving complex feedback loops. Time-lagged analyses and clear hypotheses regarding temporal sequences can help address this ambiguity. MR aims to address confounding but may not fully account for all forms, such as horizontal pleiotropy or genetic confounding. Robust MR methods like MR-Egger can help detect and adjust for these confounders. Generalizability is a concern, as MR studies often rely on specific populations. Validation in diverse populations enhances external validity. Data availability is another challenge, as MR studies depend on existing data sources. Leveraging comprehensive databases and transparent reporting can help mitigate this limitation.

From a clinical perspective, the identification of worry and anxiety as potential acute triggers for ICH bears significant implications for preventive medicine. This insight could catalyze the development of targeted interventions, including cognitive-behavioral therapy, mindfulness-based stress reduction, or pharmacological treatments. Such approaches hold promise in ameliorating the impact of negative emotional states on ICH risk, thereby offering a novel avenue for personalized healthcare. Furthermore, our findings extend to broader implications for public health, informing the design of regimen and educational programs aimed at emphasizing the importance of emotional wellbeing in stroke prevention. By elucidating the connection between mental health and stroke, heightened awareness and early interventions may be fostered, potentially reducing the incidence of ICH. Additionally, understanding the causal relationship between emotional traits and ICH stands to influence clinical practice by advocating for the integration of mental health assessments into routine care for at-risk stroke patients. This integrated approach holds potential for enhancing early detection and intervention strategies. In summary, the clinical and public health implications of our study are multifaceted. They have the potential to instigate a paradigm shift in the prevention and management of ICH, underscoring the necessity of holistic approaches that address both physical and emotional health.

## Conclusion

In conclusion, our Mendelian Randomization investigation furnishes robust evidence supporting a causal nexus between emotional dispositions of worry and anxiety and the onset of ICH. This seminal finding augments the existing paradigm, which has predominantly focused on traditional risk factors, by introducing acute emotional triggers as a crucial element in the etiology of ICH. While acknowledging the constraints of our study, we posit that our results serve as an impetus for future multi-disciplinary research aimed at elucidating the complex interplay between emotional states and cardiovascular pathologies in the context of ICH. Ultimately, our insights could inform the development of more sophisticated risk stratification algorithms and targeted interventions, thereby enriching the armamentarium for comprehensive ICH management.

## Data Availability

The datasets presented in this study can be found in online repositories. The names of the repository/repositories and accession number(s) can be found in the article/Supplementary Material.

## References

[B1] Aalbaek F. S. Graff S. Vestergaard M. (2017). Risk of stroke after bereavement-a systematic literature review. Acta Neurol. Scand. 136 (4), 293–297. 10.1111/ane.12736 28220473

[B2] Cordonnier C. Demchuk A. Ziai W. Anderson C. S. (2018). Intracerebral haemorrhage: current approaches to acute management. Lancet 392 (10154), 1257–1268. 10.1016/S0140-6736(18)31878-6 30319113

[B3] Elkind M. S. (2007). Why now? Moving from stroke risk factors to stroke triggers. Curr. Opin. Neurol. 20 (1), 51–57. 10.1097/WCO.0b013e328012da75 17215689

[B4] Fujii Y. Tanaka R. Takeuchi S. Koike T. Minakawa T. Sasaki O. (1994). Hematoma enlargement in spontaneous intracerebral hemorrhage. J. Neurosurg. 80 (1), 51–57. 10.3171/jns.1994.80.1.0051 8271022

[B5] Haynes M. (2011). Risk of vertebrobasilar stroke and chiropractic care: results of a population based case control and case-crossover study. Spine (Phila Pa 1976) 36 (1), 92. author reply. 10.1097/BRS.0b013e318200478f 21192225

[B6] Koton S. Tanne D. Bornstein N. M. Green M. S. (2004). Triggering risk factors for ischemic stroke: a case-crossover study. Neurology 63 (11), 2006–2010. 10.1212/01.wnl.0000145842.25520.a2 15596741

[B7] Krishnamurthi R. V. Feigin V. L. Forouzanfar M. H. Mensah G. A. Connor M. Bennett D. A. (2013). Global and regional burden of first-ever ischaemic and haemorrhagic stroke during 1990-2010: findings from the Global Burden of Disease Study 2010. Lancet Glob. Health 1 (5), e259–e281. 10.1016/S2214-109X(13)70089-5 25104492 PMC4181351

[B8] Larsson S. C. Burgess S. Michaelsson K. (2019). Smoking and stroke: a mendelian randomization study. Ann. Neurol. 86 (3), 468–471. 10.1002/ana.25534 31237718 PMC6701987

[B9] Lin Z. Deng Y. Pan W. (2021). Combining the strengths of inverse-variance weighting and Egger regression in Mendelian randomization using a mixture of regressions model. PLoS Genet. 17 (11), e1009922. 10.1371/journal.pgen.1009922 34793444 PMC8639093

[B10] Liu J. Luo C. Hu C. Guo Y. Cao F. Li Y. (2023). Behavioral trigger factors for hemorrhagic stroke: a case-crossover study. Postgrad. Med. J. 99 (1175), 1013–1019. 10.1093/postmj/qgad038 37209147

[B11] Lovelock C. E. Molyneux A. J. Rothwell P. M. Oxford Vascular S. (2007). Change in incidence and aetiology of intracerebral haemorrhage in Oxfordshire, UK, between 1981 and 2006: a population-based study. Lancet Neurol. 6 (6), 487–493. 10.1016/S1474-4422(07)70107-2 17509483

[B12] Maclure M. (1991). The case-crossover design: a method for studying transient effects on the risk of acute events. Am. J. Epidemiol. 133 (2), 144–153. 10.1093/oxfordjournals.aje.a115853 1985444

[B13] Maclure M. Mittleman M. A. (2000). Should we use a case-crossover design? Annu. Rev. Public Health 21, 193–221. 10.1146/annurev.publhealth.21.1.193 10884952

[B14] Mittleman M. A. Maclure M. Sherwood J. B. Mulry R. P. Tofler G. H. Jacobs S. C. (1995). Triggering of acute myocardial infarction onset by episodes of anger. Determinants of Myocardial Infarction Onset Study Investigators. Circulation 92 (7), 1720–1725. 10.1161/01.cir.92.7.1720 7671353

[B15] O'Donnell M. J. Xavier D. Liu L. Zhang H. Chin S. L. Rao-Melacini P. (2010). Risk factors for ischaemic and intracerebral haemorrhagic stroke in 22 countries (the INTERSTROKE study): a case-control study. Lancet 376 (9735), 112–123. 10.1016/S0140-6736(10)60834-3 20561675

[B16] Prasad M. Khanna P. Katyal V. K. Verma R. (2020). Acute psychological stress is a trigger for stroke: a case-crossover study. J. Stroke Cerebrovasc. Dis. 29 (6), 104799. 10.1016/j.jstrokecerebrovasdis.2020.104799 32249204

[B17] Puy L. Parry-Jones A. R. Sandset E. C. Dowlatshahi D. Ziai W. Cordonnier C. (2023). Intracerebral haemorrhage. Nat. Rev. Dis. Prim. 9 (1), 14. 10.1038/s41572-023-00424-7 36928219

[B18] Qureshi A. I. Mendelow A. D. Hanley D. F. (2009). Intracerebral haemorrhage. Lancet 373 (9675), 1632–1644. 10.1016/S0140-6736(09)60371-8 19427958 PMC3138486

[B19] Ruiz-Sandoval J. L. Cantu C. Barinagarrementeria F. (1999). Intracerebral hemorrhage in young people: analysis of risk factors, location, causes, and prognosis. Stroke 30 (3), 537–541. 10.1161/01.str.30.3.537 10066848

[B20] Scholz F.-W. (1978). Weighted median regression estimates. Ann. Statistics 6 (3), 603–609. 10.1214/aos/1176344204

[B21] Schrag M. Kirshner H. (2020). Management of intracerebral hemorrhage: JACC focus seminar. J. Am. Coll. Cardiol. 75 (15), 1819–1831. 10.1016/j.jacc.2019.10.066 32299594

[B22] Sheth K. N. (2022). Spontaneous intracerebral hemorrhage. N. Engl. J. Med. 387 (17), 1589–1596. 10.1056/NEJMra2201449 36300975

[B23] Shi Z.-M. Jing J.-J. Xue Z.-J. Chen W.-J. Tang Y.-B. Chen D.-J. (2023). Stellate ganglion block ameliorated central post-stroke pain with comorbid anxiety and depression through inhibiting HIF-1α/NLRP3 signaling following thalamic hemorrhagic stroke. J. Neuroinflammation 20 (1), 82. 10.1186/s12974-023-02765-2 36944982 PMC10031944

[B24] Skrivankova V. W. Richmond R. C. Woolf B. A. R. Yarmolinsky J. Davies N. M. Swanson S. A. (2021). Strengthening the reporting of observational studies in epidemiology using mendelian randomization: the STROBE-MR statement. JAMA 326 (16), 1614–1621. 10.1001/jama.2021.18236 34698778

[B25] Smyth A. O'Donnell M. Hankey G. J. Rangarajan S. Lopez-Jaramillo P. Xavier D. (2022). Anger or emotional upset and heavy physical exertion as triggers of stroke: the INTERSTROKE study. Eur. Heart J. 43 (3), 202–209. 10.1093/eurheartj/ehab738 34850877 PMC10503880

[B26] Smyth A. O'Donnell M. Lamelas P. Teo K. Rangarajan S. Yusuf S. (2016). Physical activity and anger or emotional upset as triggers of acute myocardial infarction: the INTERHEART study. Circulation 134 (15), 1059–1067. 10.1161/CIRCULATIONAHA.116.023142 27753614

[B27] Stalnikowicz R. Tsafrir A. (2002). Acute psychosocial stress and cardiovascular events. Am. J. Emerg. Med. 20 (5), 488–491. 10.1053/ajem.2002.34788 12216051

[B28] Thanassoulis G. O'Donnell C. J. (2009). Mendelian randomization: nature's randomized trial in the post-genome era. JAMA 301 (22), 2386–2388. 10.1001/jama.2009.812 19509388 PMC3457799

[B29] Titova O. E. Michaelsson K. Larsson S. C. (2020). Sleep duration and stroke: prospective cohort study and mendelian randomization analysis. Stroke 51 (11), 3279–3285. 10.1161/STROKEAHA.120.029902 32895015 PMC7587241

[B30] Verbanck M. Chen C. Y. Neale B. Do R. (2018). Detection of widespread horizontal pleiotropy in causal relationships inferred from Mendelian randomization between complex traits and diseases. Nat. Genet. 50 (5), 693–698. 10.1038/s41588-018-0099-7 29686387 PMC6083837

[B31] Wang Z. Wang Y. Xiong J. Gan X. Bao Y. Jiang A. (2023). Causal effects of hypertension on risk of erectile dysfunction: a two-sample Mendelian randomization study. Front. Cardiovasc Med. 10, 1121340. 10.3389/fcvm.2023.1121340 37025676 PMC10070976

[B32] Xiao L. Zou X. Liang Y. Wang Y. Zeng L. Wu J. (2022). Evaluating the causal effects of TIMP-3 on ischaemic stroke and intracerebral haemorrhage: a mendelian randomization study. Front. Genet. 13, 838809. 10.3389/fgene.2022.838809 35444693 PMC9015162

[B33] Yu Z. Zhang L. Zhang G. Xia K. Yang Q. Huang T. (2022). Lipids, apolipoproteins, statins, and intracerebral hemorrhage: a mendelian randomization study. Ann. Neurology 92 (3), 390–399. 10.1002/ana.26426 35655417

[B34] Zhu W. Gao Y. Wan J. Lan X. Han X. Zhu S. (2018). Changes in motor function, cognition, and emotion-related behavior after right hemispheric intracerebral hemorrhage in various brain regions of mouse. Brain Behav. Immun. 69, 568–581. 10.1016/j.bbi.2018.02.004 29458197 PMC5857479

